# Pharmacokinetics of Intravenously (DIZ101), Subcutaneously (DIZ102), and Intestinally (LCIG) Infused Levodopa in Advanced Parkinson Disease

**DOI:** 10.1212/WNL.0000000000200804

**Published:** 2022-09-06

**Authors:** Filip Bergquist, Mats Ehrnebo, Dag Nyholm, Anders Johansson, Fredrik Lundin, Per Odin, Per Svenningsson, Fredrik Hansson, Leif Bring, Elias Eriksson, Nil Dizdar

**Affiliations:** From the Department of Pharmacology (F.B., E.E.), University of Gothenburg; Neurology (F.B.), Sahlgrenska University Hospital, Göteborg; Department of Pharmaceutical Biosciences (M.E.), Uppsala University; Pharm Assist Sweden AB (M.E.), Uppsala; Department of Neuroscience (D.N.), Uppsala University; Department of Clinical Neurosciences (A.J., P.S.), Karolinska Institutet (A.J., P.S.), Solna; Department of Biomedical and Clinical Sciences (F.L., N.D.), Linköping University (F.L., N.D.); Neurology (P.O.), Department of Clinical Sciences, Lund University; CTC Clinical Trial Consultants AB (F.H.), Uppsala; and Dizlin Pharmaceuticals (L.B.) Gothenburg, Sweden.

## Abstract

**Background and Objectives:**

Intestinal levodopa/carbidopa gel infusion (LCIG) is superior to oral treatment in advanced Parkinson disease. The primary objective of this trial was to investigate whether continuous subcutaneous or intravenous infusion with a continuously buffered acidic levodopa/carbidopa solution yields steady-state plasma concentrations of levodopa that are equivalent in magnitude, and noninferior in variability, to those obtained with LCIG in patients with advanced Parkinson disease.

**Methods:**

A concentrated acidic levodopa/carbidopa (8:1) solution buffered continuously and administered intravenously (DIZ101) or subcutaneously (DIZ102) was compared with an approved LCIG in a randomized, 3-period crossover, open-label, multicenter trial. Formulations were infused for 16 hours to patients with Parkinson disease who were using LCIG as their regular treatment. Patients were recruited from several university neurology clinics but came to the same phase I unit for treatment. Pharmacokinetic variables and safety including dermal tolerance are reported. The primary outcomes were bioequivalence and noninferior variability of DIZ101 and DIZ102 vs LCIG with respect to levodopa plasma concentrations.

**Results:**

With dosing adjusted to estimated bioavailability, DIZ101 and DIZ102 produced levodopa plasma levels within standard bioequivalence limits compared with LCIG in the 18 participants who received all treatments. Although the levodopa bioavailability for DIZ102 was complete, it was 80% for LCIG. Therapeutic concentrations of levodopa were reached as quickly with subcutaneous administration of DIZ102 as with LCIG and remained stable throughout the infusions. Owing to poor uptake of LCIG, carbidopa levels in plasma were higher with DIZ101 and DIZ102 than with the former. All individuals receiving any of the treatments (n = 20) were included in the evaluation of safety and tolerability. Reactions at the infusion sites were mild and transient.

**Discussion:**

It is feasible to rapidly achieve high and stable levodopa concentrations by means of continuous buffering of a subcutaneously administered acidic levodopa/carbidopa-containing solution.

**Trial Registration Information:**

ClinicalTrials.gov identifier: NCT03419806. Registration first posted on February 5, 2018, first patient enrolled on February 16, 2018.

The most effective treatment of Parkinson disease is the dopamine precursor levodopa^1^ in combination with a peripheral dopa-decarboxylase inhibitor, for example, carbidopa, which reduces peripheral side effects of dopamine and increases the amount of levodopa reaching the brain. Within approximately 6 years of disease,^2^ oral levodopa treatment is however usually marred by motor fluctuations such as reoccurrence of symptoms between doses and hyperkinetic dyskinesia when levodopa concentrations are high or unstable (on-off).^3,4^

The short half-life of levodopa contributes to the development of motor fluctuations by yielding markedly fluctuating blood levels that are further aggravated by unreliable gastric emptying caused by autonomic dysfunction.^5^ In early disease, the effects of fluctuating delivery of levodopa on the brain appears to be effectively balanced by its uptake into the remaining dopaminergic neurons where dopamine can be stored until physiologically released. With progressing degeneration, however, fluctuating blood levels instead appear to cause nonphysiologic fluctuations in extracellular dopamine levels, which because of plastic changes in basal ganglia networks^6^ can result in hyperkinetic dyskinesia and off-periods.

The role of blood levodopa fluctuations for on-off was confirmed in 1975^7^ by a report showing symptom improvement with continuous intravenous administration. The poor solubility of levodopa at a physiologically acceptable pH^8^ has however hindered the clinical implementation of a concentrated levodopa/carbidopa solution feasible for parenteral infusion. Proof of the clinical usefulness of continuous levodopa administration in outpatients has instead been obtained using a levodopa/carbidopa-containing gel administered intestinally (LCIG; Duodopa), which displays superiority over oral treatment^9-12^ with at least 2 hours of additional daily ON-time without troublesome dyskinesia. However, LCIG requires gastric surgery and is associated with considerable risk for device complications.^11,13,14^

More practical than intestinal administration would be to use a subcutaneous route, as is currently done with the dopamine agonist apomorphine. Therefore, the purpose of this study was to explore whether a complete and rapid uptake of levodopa from the subcutaneous tissue may be obtained by the administration of a recently developed levodopa (10 mg/mL) plus carbidopa (1.25 mg/mL) solution (DIZ102) consisting of a stock solution where levodopa and carbidopa are dissolved at a low pH brought to a physiologically favorable pH (5.0–5.3) by continuous mixing with a buffer. Patients with Parkinson disease and chronic LCIG treatment were hence studied at 2 occasions during which they received intestinal administration of LCIG and subcutaneous administration of DIZ102, respectively, for 16 hours. At a third occasion, levodopa/carbidopa was administered intravenously using DIZ101, which differs from DIZ102 in the composition of the buffer, but not the stock solution.

## Methods

### Participants and Study Design

This was a randomized, 3-period crossover, open-label, multicenter trial. Patients with Parkinson disease and ongoing stable LCIG treatment (600 mg–4,000 mg levodopa per day) for at least 30 days before screening were eligible to participate provided that their the Hoehn and Yahr score was ≤3 during LCIG infusion and their body mass index was between 18.0 and 35.0 kg/m^2^. Patient recruitment and end of study safety follow-up were undertaken at 5 university neurology clinics in Sweden, but all study treatments and evaluations were performed at the same phase I clinical trial unit, Gothia Forum CTC at Sahlgrenska University Hospital, in Gothenburg, Sweden, between June 2019 and March 2020. The patients reported here were included after a major change of protocol after an interim analysis of 5 patients who had been enrolled in 2018 under previous protocol. Participants received 3 treatments in a randomized order: intestinal infusion of LCIG (Duodopa; Fresenius Kabi Norge AS, Halden, Norway), intravenous infusion of DIZ101, and subcutaneous infusion of DIZ102. Each treatment was preceded by levodopa abstinence for at least 8 hours and followed by a time to next visit of at least 3 days during which the patients were on their regular LCIG regimen. At each treatment occasion, repeated plasma samples for pharmacokinetic analyses were collected for 24 hours.

### Objectives

The primary objective was to demonstrate that DIZ101 and DIZ102 yield steady-state plasma concentrations of levodopa equivalent to those obtained with LCIG with a variability in plasma concentrations noninferior to that obtained with LCIG. Secondary objectives included safety, other pharmacokinetic variables, and motor outcome. The registered primary outcome variables were AUC for levodopa vs time and coefficient of variation (COV) for plasma levodopa concentrations. A one-sided 90% CI was used to define noninferiority of COV. Secondary registered variables were skin tolerance, bioavailability of levodopa and carbidopa, C_max_, t_max_, and t_½_ for levodopa and carbidopa, and AUC for carbidopa vs time. Motor symptom ratings using the Treatment Response Scale and objective accelerometry (Parkinson KinetiGraph) were secondary outcomes that will be reported elsewhere. In addition to the registered variables, post hoc analyses regarding peak-to-trough variability and detrended COV for levodopa plasma concentration were undertaken.

### Treatments and Dosing

DIZ101 and DIZ102 were supplied in 2 bottles, one with a concentrated levodopa/carbidopa solution (20 and 2.5 mg/mL, respectively) and one with a buffer. The solutions were mixed 1:1 continuously during infusion, the infused products thus having a final concentration of 10 mg/mL of levodopa and 1.25 mg/mL of carbidopa. DIZ101 was infused through an indwelling vein catheter in the arm and DIZ102 through 2 subcutaneous infusion catheters (Cleo 90; Smiths Medical, MN) at the abdomen after a split of the infusion line after mixing (eFigure 1, links.lww.com/WNL/C133). DIZ101 and DIZ102 were delivered using 2 Braun SPACE Infusion Pumps (B. Braun Melsungen AG, Melsungen, Germany). LCIG was delivered using the patient's regular pump (CADD-Legacy Duodopa; Smiths Medical, MN) and the implanted percutaneous endoscopic transgastric jejunostomy system.

Study treatments were administered as a morning bolus dose followed by an infusion with a fixed flow rate for the rest of the treatment period. In each patient, the dosing of the 3 treatments was based on their regular prestudy hourly doses of LCIG during continuous infusion defined as the postbolus infusion rate (mg/h) plus the regular total amount of levodopa extra doses (mg) divided by the regular infusion time (h). The total doses of DIZ101 and DIZ102 were based on bioavailability data from 5 patients who were enrolled before an interim analysis^15^ that led to a modification of the dosing and composition of the buffer; these 5 patients are therefore not included in the results presented here. The interim analysis had indicated that the levodopa bioavailability be 82% with LCIG and 95% with DIZ102 using DIZ101 as reference; the equivalent levodopa doses for DIZ101 and DIZ102 were hence calculated to be 81% and 86% of that of LCIG, respectively. For LCIG, the effective morning bolus dose was 110% of the hourly infusion dose plus 3 mL to fill the duodenal tube (flow rate: 800 mg/h; 40 mL/h). For DIZ101, the bolus was also 110% of the hourly dose (600 mg/h; 60 mL/h). Because the pilot study had revealed a somewhat slower initial levodopa uptake of DIZ102, the bolus dose for DIZ102 was higher—155% of the hourly DIZ102 dose—and infused at a rate of 800 mg/h (80 mL/h). Additional bolus doses and changes in flow rates were only allowed if medically necessary.

### Pharmacokinetic Evaluation

Blood samples for pharmacokinetic evaluation were drawn short before the start of treatment and at 0, 0.25, 0.5, 1, 1.5, 2, 2.5, 3, 3.5, 4, 5, 6, 7, 8, 10, 12, 14, 16, 16.5, 17, 17.5, 18, 23, and 24 hours relative to the start of the bolus infusion. Levodopa, carbidopa, and the levodopa metabolite 3-O-methyldopa (3-OMD) were analyzed in plasma using ultra-performance liquid chromatography-tandem mass spectrometry, with the lower limit of quantification (LLOQ) being 15 ng/mL for levodopa and carbidopa and 187.5 ng/mL for 3-OMD.

We analyzed (1) maximum observed concentration (C_max_), (2) time to C_max_ (T_max_), (3) area under the curve (AUC) for different time intervals reflecting both overall and early and late exposure, (4) concentration before administration (C_0_), (5) last determinable concentration (C_last_) and AUC_0–last_, (6) elimination half-life (t_1/2_), (7) AUC_0–∞_, (8) AUC_0–∞_/dose (dosage normalized AUC_0–∞_) for levodopa and carbidopa, and (9) fluctuations assessed as COV and peak-to-trough fluctuation (PTF) including analysis of detrended COV to separate rapid fluctuations from long-term trends for levodopa. If baseline plasma concentrations of levodopa, carbidopa, or 3-OMD were higher than LLOQ, a baseline elimination curve was calculated and subtracted from the actual plasma concentrations, assuming that the elimination rates would be the same as after the LCIG treatment for the participant in question.

### Safety Evaluation

Safety variables included standard laboratory tests at screening and after the third treatment. To address the possibility that the citrate component of the buffers would have a systemic effect on coagulation by binding calcium, or influence acid-base balance systemically, measurement of P-Mg^2+^, P-Ca^2+^, ionized Ca^2+^, and base excess was undertaken before and on 4 occasions during infusion of each treatment. Adverse events were evaluated during and after treatment. Infusion sites were photographed before and at 24 hours and blindly assessed by 2 dermatologists in a randomized order and rated using the Draize skin reaction scale.^16^

A follow-up examination of subcutaneous infusion sites was performed 4 weeks after the DIZ102 treatment. A safety follow-up visit at the patient's local clinic was performed 4 weeks after the last treatment.

### Randomization and Masking

A permuted block randomization with a block size of 6 and equal distributions of the 6 possible treatment orders was undertaken using a pseudorandom generator (SAS Institute). Participants were allocated to the respective treatment sequence at the first treatment visit using sealed envelopes. Masking of treatment during video recordings of motor performance was achieved using dummy infusion lines and pumps.

### Statistical Analysis

A power analysis indicated that n = 14 would be sufficient for establishing dose-adjusted bioequivalence (2-sided CI) and n = 18 for ascertaining noninferiority (one-sided CI) about COV. This was based on a similar study regarding 16-hour intestinal infusion of LCIG^17^ and data from the 5 patients assessed in the interim analysis that led to a restart of this trial.

To compare the 3 treatments about levodopa and carbidopa levels, including the initial phase of increasing levels and the decline after the termination of infusion, comparisons of levodopa and carbidopa AUC_0–last_, AUC_0–∞_, AUC_0–16_, AUC_2–16_, AUC_0–2_, and AUC_8–16_ (subscript: hours) were undertaken using the analysis of variance (ANOVA) of logarithmic values with the resulting point estimates and CIs being reported after back-transformation to nominal values. The terms in the ANOVA were sequence, patient within sequence, period, and treatment. To compare levodopa and carbidopa systemic exposure, standard average bioequivalence testing based on the 90% CI for the ratio of the population means (test/reference) was used. The acceptance range for the AUC ratio of the 90% CI for levodopa was 0.8000–1.2500. Bioequivalence tests were undertaken for DIZ101 and DIZ102 vs LCIG (reference) and for DIZ102 and LCIG vs DIZ101 (reference).

The one-sided 90% CI noninferiority test of the COV for levodopa levels was calculated for DIZ101 and DIZ102 vs LCIG (reference) and for DIZ102 and LCIG vs DIZ101 (reference). In addition, it was assessed whether there were significant differences in COV between treatments using ANOVA after logarithmic transformation with the terms sequence, patient within sequence, period, and treatment. Furthermore, to remove the effect of long-term trends on the assessment of short-term variability, a post hoc detrended analysis of short-term fluctuations during the 2-hour to 16-hour period was performed by constructing a time series based on the difference between the original observation and that of the previous time point.

To calculate how fast the 3 treatments achieved 50 or 90% of the individual 2-hour to 8-hour mean levodopa plasma concentration obtained with LCIG, the individual PK profile for LCIG was used to determine the mean concentration over 2–8 hours by dividing AUC 2–8 hours with 6. The time when the plasma concentration reached 50% and 90% of the mean LCIG concentration between 2 and 8 hours was determined by assuming a linear change in plasma concentration between each measurement and calculating the time when the linear extrapolation first reached these values.

Other reported comparisons of pharmacokinetic data were performed with paired Student *t*-tests. *p* Values of 0.05 or less were considered significant.

### Standard Protocol Approvals, Registrations, and Patient Consents

The study was conducted in accordance with the International Conference on Harmonization Good Clinical Practice guidelines and the Declaration of Helsinki and approved by the Swedish Ethical Review Authority (658-17) and the Swedish Medical Products Agency (EudraCT: 2017-002488-17). All participants provided written informed consent. The trial was registered at ClinicalTrials.gov: NCT03419806. The original protocol (IPO001 v4.1) contained an interim analysis after the first 5 patients, which has been published as a poster abstract.^15^ This analysis prompted a modification of the dosing schedule, the buffer composition for DIZ102, and the primary outcome variables leading to a restart of the trial after which the 20 patients presented in this report were enrolled. The study protocol (IPO001 v5.1) and statistical analysis plan are available in eSAP 1 and eSAP 2 (links.lww.com/WNL/C134 and links.lww.com/WNL/C135).

### Data Availability

The anonymized patient data and related clinical trial study documents are not being publicly shared as long as they are part of an ongoing or planned regulatory submission. Anonymized pharmacokinetic data can be provided to qualified researchers after approval of the research proposal.

## Results

### Patient Characteristics

Twenty-two patients were screened, and 20 were included and randomized. Two patients who discontinued after the first treatment (in both cases DIZ101) were excluded from the pharmacokinetic analyses (n = 18) but included in the tolerability assessments (n = 20). The median age of the 20 included patients was 68.5 years (range 46–77 years), 60% were men, and the median body mass index was 24.3 kg/m^2^ (range 19–35 kg/m^2^). The median LCIG-derived prestudy daily levodopa dose in patients who completed all treatments (n = 18) was 1,156 mg (range 641–2,205 mg). Possible add-on treatments containing oral levodopa, a decarboxylase inhibitor, or entacapone were withheld for >8 hours before the start of the administration of trial medication and for the following 24 hours. Other PD treatments, including dopamine agonists, monoaminoxidase B inhibitors, and amantadine were allowed throughout the study.

### Levodopa Levels

**Figure 1:**
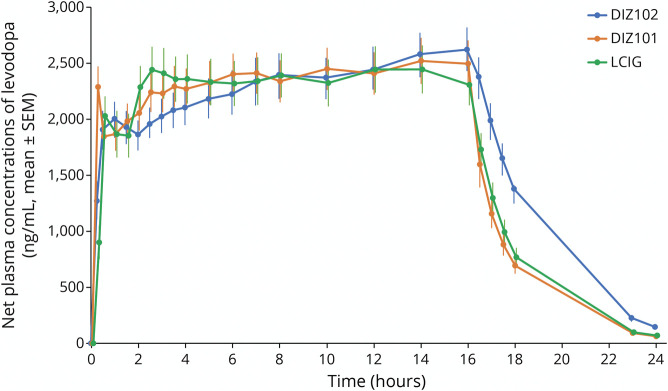
Net Plasma Concentrations of Levodopa (ng/mL, mean ± SEM) Before, During 16-Hour Infusion, and up to 24 Hours After Infusion Start With the 3 Treatments DIZ101 (Intravenous), DIZ102 (Subcutaneous), and LCIG (Intestinal)

The intravenous DIZ101 bolus dose resulted in higher plasma concentrations at t = 15 minutes compared with that after LCIG (*p* < 0.0001) or DIZ102 (*p* = 0.0002, Figure 1). After 30 minutes, there were no differences between treatments (Figure 1), which all reached mean net values of about 2000 ng/mL within 1–2 hours; after this, they displayed a further increase, with LCIG appearing to reach a plateau at approximately 3 hours, DIZ101 at approximately 5 hours, and DIZ102 at approximately 7–8 hours. After the termination of the infusion, the decline in plasma levels was somewhat slower after subcutaneous administration with significant differences in t_1/2_ for DIZ102 vs LCIG (*p* = 0.02), but not for DIZ102 vs DIZ101 (*p* = 0.09) or DIZ101 vs LCIG (*p* = 0.11 using logarithmic values; Table 1).

**Table 1:**
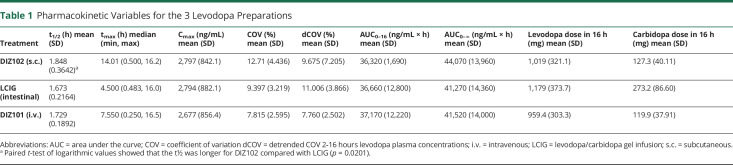
Pharmacokinetic Variables for the 3 Levodopa Preparations

### Bioavailability

With dose adjustments compensating for the poorer bioavailability of LCIG, and somewhat slower uptake of DIZ102, both DIZ101 and DIZ102 met levodopa bioequivalence criteria vs LCIG for AUC (including partial AUCs) and C_max_ comparisons (Table 2).

**Table 2:**
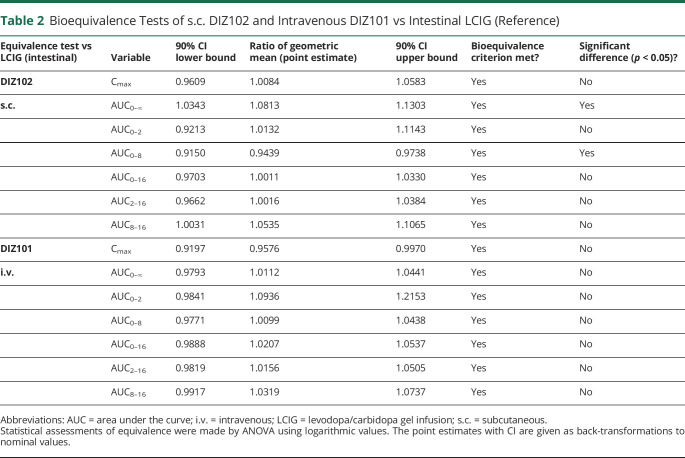
Bioequivalence Tests of s.c. DIZ102 and Intravenous DIZ101 vs Intestinal LCIG (Reference)

With DIZ101 as reference and based on AUC_0-∞_, the bioavailability of levodopa was 101% (97%–104%, CI_90%_) with DIZ102 and 80% (78%–83%, CI_90%_) with LCIG.

### Fluctuations in Levodopa Levels

**Figure 2:**
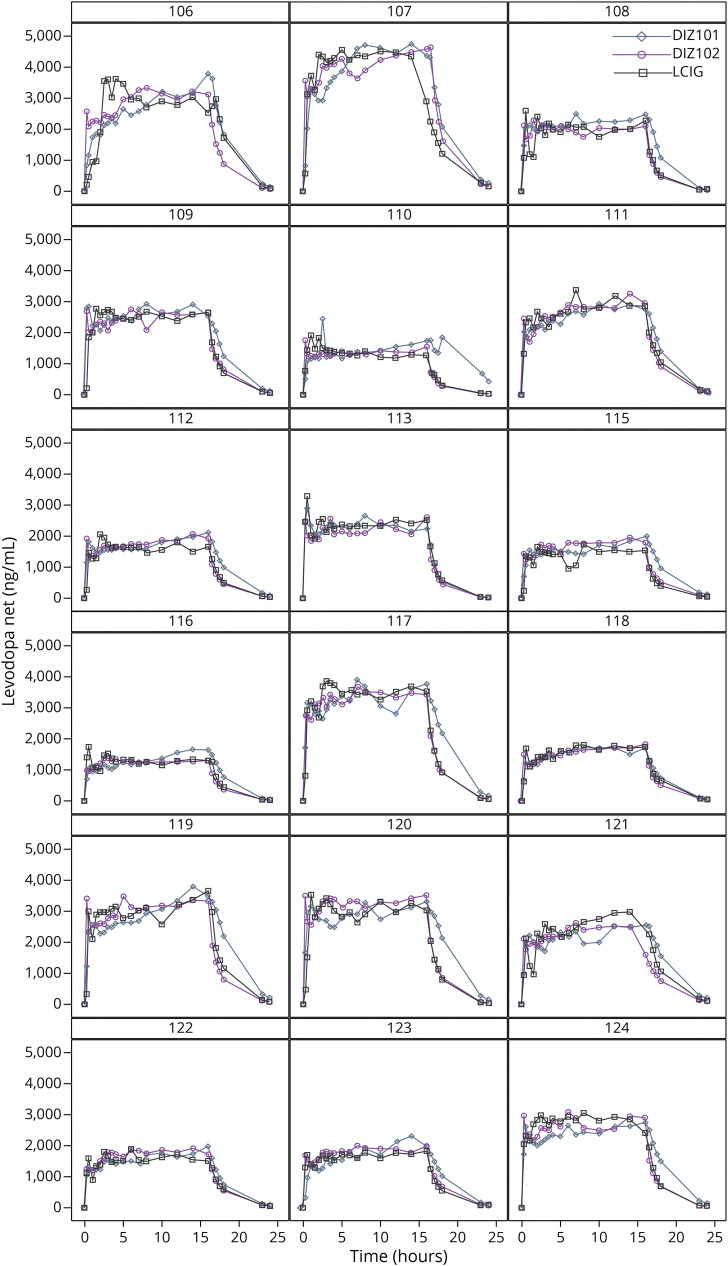
Individual Plasma Levodopa Concentration Time Profiles (ng/mL, 0–24 Hours) for the Three 16-Hour Infusions With DIZ101 (Intravenous), DIZ102 (Subcutaneous), and LCIG (Intestinal)

The COV of DIZ102 for levodopa levels was noninferior to that of LCIG, with the lower 90% CI bound below 1.2500 at 1.2254, but ANOVA indicated that COV was lower with LCIG than with DIZ102 (*p* = 0.0004, Table 3). However, inspection of individual plasma curves (Figure 2) suggested that the higher variability with DIZ102 levodopa concentrations was not caused by rapid fluctuations but by a different slope of the 2-hour to 16-hour curve. A detrended analysis of levodopa concentration COV hence undertaken again showed that COV DIZ102 was noninferior to LCIG. DIZ101 displayed significantly lower detrended COV than LCIG (Table 3) but did not differ significantly from DIZ102.

**Table 3:**
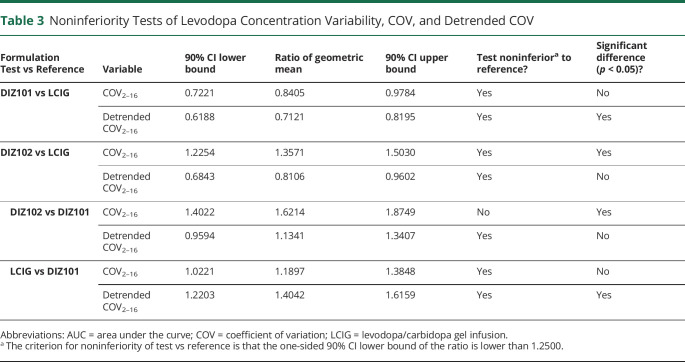
Noninferiority Tests of Levodopa Concentration Variability, COV, and Detrended COV

Similar to COV, the lowest values of PTF over 2–16 h were found with DIZ101 (0.27 ± 0.08; mean ± SD), followed by LCIG (0.34 ± 0.13) and DIZ102 (0.42 ± 0.16) (LCIG vs DIZ102: *p* = 0.04; DIZ101 vs DIZ102: *p* < 0.001; and LCIG vs DIZ101: *p* = 0.09, using logarithmic PTF).

With LCIG, the time to reach 50% and 90% of the LCIG steady-state concentrations of levodopa was 22 and 60 minutes, respectively, including a mean time of 4.5 minutes to fill the intestinal tube. DIZ101 reached 50% and 90% in 8 and 40 minutes, respectively; DIZ102 reached 50% in 17 minutes and 90% after 2.5 hours.

### Plasma Carbidopa Levels

**Figure 3:**
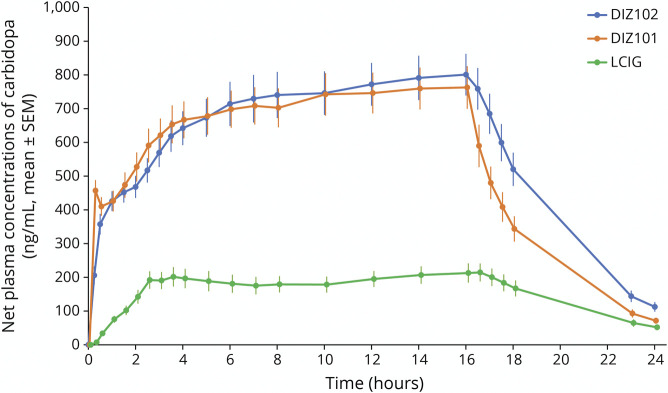
Net Plasma Concentrations of Carbidopa (ng/mL, Mean ± SEM) Before, During 16-Hour Infusion, and up to 24 Hours After Infusion Start With the 3 Treatments DIZ101 (Intravenous), DIZ102 (Subcutaneous), and LCIG (Intestinal)

The bioavailability of carbidopa based on dose-adjusted AUC_0–∞_ with DIZ101 as reference was 102% (98%–106%, CI_90%_) for DIZ102 but only 12% (11%–14%, CI_90%_) for LCIG. Despite a lower ratio of anhydrous carbidopa to levodopa in DIZ101 and DIZ102 (1:8) than in LCIG (1:4.3), plasma concentrations of carbidopa were hence approximately 4 times higher with the former than with the latter (Figure 3).

### Plasma Concentrations of 3-OMD

3-OMD is a major metabolite of levodopa produced by the catechol-O-methyl-transferase and has a half-life of >12 hours.^18^ Baseline 3-OMD concentration before infusion was significantly lower on the day of DIZ102 administration suggesting incidentally lower levodopa exposure in the days preceding this treatment. An analysis of a calculated elimination slope of net values obtained by removing the baseline value revealed a longer 3-OMD t_½_ for DIZ102 than for the other administrations (*p* = 0.002 vs LCIG and *p* < 0.001 vs DIZ101). T_max_ of 3-OMD was 17.0 hours for all 3 treatments. C_max_ was 7,344 ng/mL, 7,133 ng/mL, and 7,072 ng/mL for DIZ102, LCIG, and DIZ101, respectively.

### Safety

Safety laboratory data (hematology, clinical chemistry including homocysteine, and urine analysis) were stable throughout the study, and this was also the case for calcium, ionized calcium, magnesium, and base excess during and 8 hours after infusion. The only serious adverse event was a device complication related to a patient's regular LCIG treatment that was discovered the day after treatment with DIZ101 (Table 4).

**Table 4:**
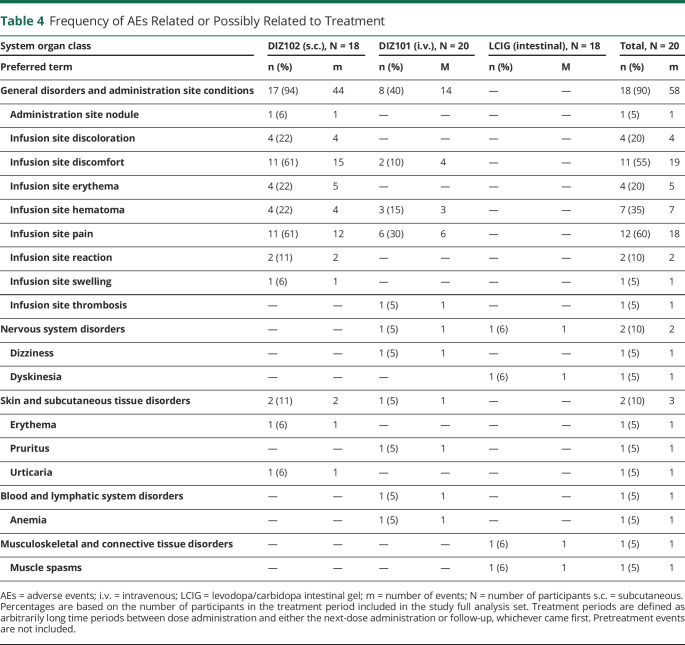
Frequency of AEs Related or Possibly Related to Treatment

### Local Tolerance

During the subcutaneous infusion of DIZ102, most participants (16/18) experienced pain or discomfort, which was often mild, sometimes moderate, and never serious, and almost exclusively reported during the bolus infusion, which had a mean duration of 7 minutes. On inquiry immediately after the bolus, all patients expressed that this experience would be fully endurable also if experienced daily. At the predefined assessment points at 2, 8, and 16 hours during the remaining treatment time, pain or tenderness was reported only by 2 patients treated with DIZ102 and rated as mild (visual analog scale ≈30/100).

Pain or discomfort during the bolus phase occurred also with intravenous administration but at a lower rate (in 6 of 18 patients). At later time points, pain or tenderness was rated as 15–30/100, which is what can be expected from peripheral intravenous catheters.

Four DIZ102-treated and 3 DIZ101-treated participants developed painless and diffuse infusion site hematomas, best described as bruising, which appeared several hours after the initiation of infusion (mean size: 3.8 × 3.8 cm for DIZ102 and 4.4 × 4.4 cm for DIZ101). They had always resolved 1 month after subcutaneous treatment. In one patient receiving DIZ102, there was a small nodule, unnoticed by the patient, at the infusion site. In one patient, thrombophlebitis, which did not require treatment, developed after intravenous infusion. For a list of adverse events, see Table 4.

Dermatologic assessments of infusion sites using Draize scores (0–8 points, normal skin = 0) indicated mild skin irritation with all treatments; with LCIG, this was related to the PEG stoma and not the actual infusion point. With LCIG, the mean (SD) baseline score was 1.3 (1.2) points and did not change after treatment. With DIZ102, Draize scores were 0.14 (0.23) before treatment and 0.88 (0.65) after treatment and with DIZ101 0.025 (0.112) before treatment and 1.23 (1.1) after treatment. Although raters were instructed to disregard marks caused by adhesive tapes, some ratings nevertheless appear to refer to such marks. Occasionally bruising first presented as erythematous discoloration and captured by the Draize rating, although Draize scores are not intended for rating hematoma.

## Discussion

The major finding of this study is that it is possible to rapidly obtain stable plasma levels of levodopa at therapeutic levels (≈2,000 ng/mL) by subcutaneous administration of a concentrated levodopa/carbidopa solution with a pH of ≈5. With dose adjustments aimed to compensate for the higher bioavailability and somewhat slower absorption of subcutaneously administered levodopa, the aims to demonstrate bioequivalence of DIZ102 vs LCIG about levodopa levels were met.

For local tolerability reasons,^19^ a levodopa/carbidopa solution aimed for subcutaneous delivery should preferably not be overly acid or alkaline. The solubility of both levodopa and carbidopa is, however, markedly pH-dependent, with high solubility in acid and alkaline solutions, but low (≈3 mg/mL) at physiologic pH. As presently shown, this problem can be circumvented by continuous buffering of an acidic levodopa/carbidopa stock solution. This stock solution is stable for >3 months in room temperature, and the buffered mixture has sufficient physical and chemical stability to enable safe administration with a considerable time margin (>2 hours, Dizlin Pharmaceuticals, data on file).

Although all 3 treatments yielded similar levodopa levels within 30 minutes, which is when the therapeutic effect of LCIG is usually experienced, intravenous DIZ101 displayed the fastest increase. The levodopa level after subcutaneous DIZ102 reached its maximum later and had a 9% longer t_½_. These differences, which appear too small to be clinically important, can probably be explained by the subcutis serving as a levodopa reservoir. Because data from the interim analysis had indicated a slower rise in levodopa plasma concentrations with DIZ102, a higher bolus was used for this treatment. The differences yet observed for the initial increase in levodopa levels might be reduced with further adjustments of the bolus size.

Because the incentive for continuous levodopa infusion is to reduce symptom fluctuations by abolishing the rapid changes in blood levels after oral administration, the treatments were also compared concerning variations in levodopa concentrations. Noninferiority of the levodopa plasma concentration COV was found for DIZ101 and DIZ102 compared with LCIG. With detrended analysis, point-to-point fluctuations were smaller with DIZ101 than with DIZ102, and with DIZ102 than with LCIG, but only the difference between DIZ101 and LCIG was statistically significant.

One reason for including intravenous delivery of levodopa in this trial was to obtain a reference for bioavailability assessments for the subcutaneous and the intestinal infusions. It also allowed us to determine the absolute levodopa bioavailability with LCIG. This analysis showed the bioavailability of levodopa to be 80% with LCIG and 101% with DIZ102 repeated information.

In line with previous studies,^20^ the systemic bioavailability of carbidopa was low (13%) with LCIG. Thus, although the carbidopa-levodopa ratio in LCIG (1:4.3) is about twice that of DIZ102 and DIZ101 (1:8), carbidopa plasma concentrations were 4 times higher with the latter treatments, for which the carbidopa bioavailability was 100%. It should be considered that the decarboxylation of levodopa to dopamine on enteral administration probably to a large extent (but not solely)^21^ takes place in the intestinal mucosa and liver; plasma levels of carbidopa in LCIG-treated patients hence should not be expected to reflect the carbidopa concentration at the place where the relevant enzyme inhibition takes place, that is, intestinally. Previous studies of subcutaneous administration of levodopa/carbidopa^22^ or foslevodopa/foscarbidopa^23^ indicate that reducing the ratio below 1:8 or 1:10, respectively, is associated with lower levodopa levels.

The systemic carbidopa levels observed are not likely of concern regarding tolerability because similar levels may occur in patients medicating with high oral levodopa/carbidopa doses^24^—a treatment that has been in clinical use for over 50 years with no evidence of side effects produced by the carbidopa molecule (apart from those caused by increasing levodopa). Moreover, it has been suggested that the conventional oral doses of carbidopa are insufficient for optimal inhibition of peripheral levodopa decarboxylation,^21,25,26^ tentatively because of an induction of the decarboxylase upon repeated inhibitor administration.^27^

Although LCIG is efficacious in reducing pharmacokinetically driven symptom fluctuations, the necessity of implanting a chronic percutaneous gastrointestinal tube, and complications related to this, constitutes limitations to this treatment modality. Subcutaneous administration, such as DIZ102, or continuous infusion of apomorphine that is available in the EU, is less invasive. With both DIZ101 and DIZ102, local discomfort or pain during the brief bolus infusion which was given at a relatively high flow rate was the most common adverse event. The reasons for bolus-related discomfort and pain are not obvious, but factors such as volume, osmolarity, and composition of the buffer may contribute. Although all patients expressed that they would find the experience of the bolus infusion fully endurable also on a daily basis, an oral loading dose of levodopa can eliminate the need for bolus infusion. Other local adverse events included bruising near the infusion site in 4 of 18 patients with DIZ102 and 3 of 18 patients with DIZ101. Bruising was modest in size and of mild severity, and erythema was short-lasting. In one patient, phlebitis developed near the intravenous infusion site and resolved spontaneously within approximately 1 week.

ND0612 is a levodopa/carbidopa solution aimed for subcutaneous administration, with the same carbidopa vs levodopa ratio as DIZ102 but differing from the latter by being more concentrated (60 mg/mL of levodopa) and displaying an alkaline pH. Although ND0612 reportedly may replace approximately 600 mg, or two-thirds of the total daily levodopa intake,^28^ this study indicates that it would be possible to fully replace at least 2,000 mg of levodopa with DIZ102.

A slightly acidic pH, as that of DIZ102, may also be more favorable than a slightly alkaline pH, as that of ND0612, considering skin tolerability. This includes the risk for noduli, which seems relatively high in patients receiving repeated administration of ND0612.^29^ Although the long-term tolerability of DIZ102 remains to be assessed, the one nodule observed in this study was small and unnoticed by the patient.

Another solution intended for subcutaneous administration is ABBV-951, which contains the prodrugs foslevodopa and foscarbidopa and appears to produce plasma levels of levodopa in healthy individuals that appear comparable with those obtained in this study.^23^ As yet, publicly available data on ABBV-951 in patients are sparse.

Although both ND0612 and ABBV-951 are intended for a 24-hour regimen, DIZ102 infusion was restricted to 16 hours in this study because this, at the time, was the approved duration for the reference, LCIG. The relatively rapid increase and decline in levodopa levels obtained with DIZ102 should enable a considerable flexibility in dosing, including both 24-hour administration and shorter treatment durations, and allowing occasional bolus dosing if required.

Nevertheless, an exposure period of merely 16 hours is insufficient to evaluate the long-term safety of daily infusions of DIZ102. Another limitation is the relative homogeneity of the studied population; there is, however, no reason to believe that the pharmacokinetics of intravenous or subcutaneous levodopa should be different in other populations. In addition, the studied population is likely representative of the group of patients where subcutaneous treatment would currently be considered.

In conclusion, this study demonstrates that subcutaneous administration of a levodopa/carbidopa solution at a pH of ≈5 may rapidly (≤30 minutes) produce stable plasma levodopa levels in blood of sufficient magnitude to enable such treatments to be used as monotherapy also for patients with Parkinson disease requiring relatively high doses.

## Glossary

3-OMD: 3-O-methyldopa
ANOVA: analysis of variance
AUC: area under the curve
COV: coefficient of variation
LCIG: levodopa/carbidopa gel infusion
LLOQ: lower limit of quantification
PTF: peak-to-trough fluctuation
